# Chronic TLR Stimulation Controls NLRP3 Inflammasome Activation through IL-10 Mediated Regulation of NLRP3 Expression and Caspase-8 Activation

**DOI:** 10.1038/srep14488

**Published:** 2015-09-28

**Authors:** Prajwal Gurung, Bofeng Li, R. K. Subbarao Malireddi, Mohamed Lamkanfi, Terrence L. Geiger, Thirumala-Devi Kanneganti

**Affiliations:** 1 Department of Immunology, St. Jude Children’s Research Hospital, Memphis, TN, 38105, USA; 2 Department of Pathology, St. Jude Children’s Research Hospital, Memphis, TN, 38105, USA; 3 Department of Medical Protein Research, VIB, B-9000 Ghent, Belgium; 4 Department of Biochemistry, Ghent University, B-9000 Ghent, Belgium

## Abstract

While the molecular mechanisms promoting activation of the Nod-like Receptor (NLR) family member NLRP3 inflammasome are beginning to be defined, little is known about the mechanisms that regulate the NLRP3 inflammasome. Acute (up to 4 hours) LPS stimulation, followed by ATP is frequently used to activate the NLRP3 inflammasome in macrophages. Interestingly, we observed that the ability of LPS to license NLRP3 is transient, as prolonged (12 to 24 hours) LPS exposure was a relatively ineffective priming stimulus. This suggests that relative to acute LPS, chronic LPS exposure triggers regulatory mechanisms to dampen NLRP3 activation. Transfer of culture supernatants from macrophages stimulated with LPS for 24 hours dramatically reduced ATP- and nigericin-induced NLRP3 inflammasome activation in naïve macrophages. We further identified IL-10 as the secreted inflammasome-tolerizing factor that acts in an autocrine manner to control activation of the NLRP3 inflammasome. Finally, we demonstrated that IL-10 dampens NLRP3 expression to control NLRP3 inflammasome activation and subsequent caspase-8 activation. In conclusion, we have uncovered a mechanism by which chronic, but not acute, LPS exposure induces IL-10 to dampen NLRP3 inflammasome activation to avoid overt inflammation.

The immune system is equipped with a set of pathogen recognition receptors (PRRs) that function to mount an immune response against invading pathogens. Toll-like receptors (TLRs) recognize pathogen associated molecular patterns (PAMPs) in the extracellular milieu and endosomes, while Nod-like receptors (NLRs) patrol the cytoplasm^1^. A set of Nod-like receptors that include NLRP1b, NLRP3 and NLRC4 assemble multi-protein complexes termed inflammasomes^2^,^3^,^4^,^5^,^6^. Inflammasome assembly is critical for activation of caspase-1, which ultimately cleaves pro-IL-1β and pro-IL-18 into their mature bioactive forms^6^. Because of their potent inflammatory activities, IL-1β and IL-18 are regulated at multiple levels^7^. Specifically, two signals are required for the activation of inflammasomes and the release of bioactive IL-1β and IL-18. The first step involves recognition of PAMPs such as LPS, PGN and polyI:C by TLRs to induce the priming signals that are necessary for upregulation of inflammasome components and pro-IL-1β. In the second step, recognition of cytoplasmic danger signals by NLRs results in assembly of the inflammasome that processes pro-IL-1β and pro-IL-18^7^,^8^. This coordinated regulation of the inflammasomes is necessary to limit unwanted inflammatory responses.

Inflammatory responses are akin to a double-edged sword and our immune system has several regulatory checkpoints to control these responses; too little and the pathogens take over or too much and the hyperinflammatory responses cause tissue damage. Aptly so, our immune system employs several negative feedback pathways to shut down inflammatory responses to avoid excessive damage to self-tissues. Once TLRs are activated by their cognate PAMPs, robust activation of NFκB and MAP kinase signaling pathways induce upregulation of several hundred genes, mostly of the proinflammatory signature^9^,^10^,^11^,^12^. Studies tracking the time course of proinflammatory cytokine mRNA expression levels has shown that these mRNAs get upregulated as early as 30 minutes post TLR stimulation, peak within a couple of hours and return to basal expression by 4–6 hours^13^,^14^. This coordinated regulation of the pro-inflammatory gene expression is necessary to avoid unwanted inflammation and several mechanisms contribute to the negative regulation of these pathways^15^.

Once stimulated with TLR ligands, macrophages become refractory to subsequent challenge with similar ligands. This phenomenon is now widely referred to as endotoxin tolerance or LPS tolerance^16^. Studies have shown that similar tolerance can be achieved with stimulation of other TLRs and NLRs such as TLR2, TLR5, TLR9 and NOD2^17^,^18^,^19^,^20^. While much is known about the negative regulation of inflammatory signaling and cytokine production during TLR stimulation, whether inflammasomes are also similarly regulated is not known. Here, we utilized a well-established LPS (1^st^ signal–priming)/ATP (2^nd^ signal- NLR activation) stimulation protocol to study activation of the NLRP3 inflammasome. Our study demonstrates that while acute LPS stimulation (4 h) induces robust NLRP3 inflammasome activation, chronic LPS stimulation (12 h–24 h) results in attenuation of the NLRP3 inflammasome. We further identify IL-10 as a soluble secreted factor that acts through a negative feedback loop to dampen NLRP3 inflammasome activation. Our studies have thus uncovered a role for IL-10 in tempering activation of the NLRP3 inflammasome in response to chronic TLR stimulation.

## Results

### Chronic TLR engagement negatively regulates NLRP3 inflammasome activation

**Figure 1:**
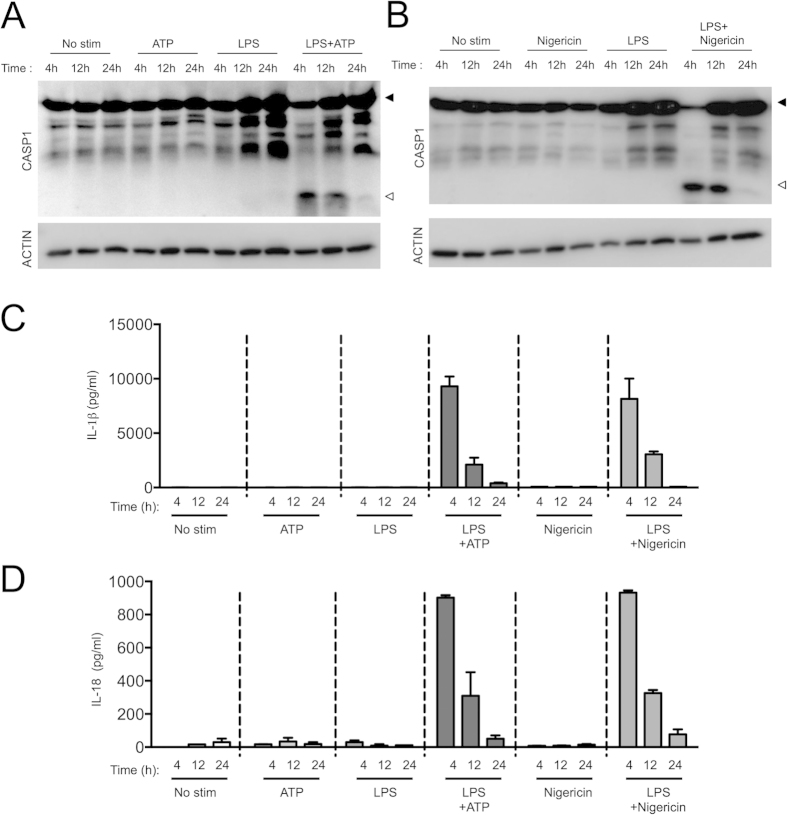
Chronic LPS stimulation weakly activates NLRP3 inflammaome activation. Wildtype (WT) BMDMs were stimulated with LPS for 4, 12 and 24 hours followed by ATP or nigericin for the last 30 minutes. (**A**,**B**) BMDMs were stimulated with LPS/ATP or LPS/Nigericin as indicated and lysates were blotted for caspase-1 and actin. Cell supernatants collected after LPS/ATP or LPS/Nigericin stimulations were analyzed by ELISA for IL-1β (**C**) and IL-18 (**D**). Solid arrow represents pro-form and open arrow represents cleaved-form of the protein in Western blots. ELISA data are presented as means ± s.e.m. of technical replicates and all data are representative of at least three independent experiments.

Prolonged LPS stimulation results in the inhibition of proinflammatory gene expression to avoid excessive inflammation^14^,^21^. Recent study by Schroder *et al.* has shown that duration of LPS priming could adversely affect the activation of caspase-1 and IL-1β by ATP and nigericin^22^. To examine whether we could recapitulate similar phenomenon during NLRP3 inflammasome activation, BMDMs were stimulated with LPS for different periods of time before being exposed to 5 mM ATP for 30 minutes (Fig. 1A). As expected, macrophages stimulated with LPS for 4 h responded to ATP treatment with robust caspase-1 activation as evident by the appearance of the activation-associated 20 kDa (p20) fragment in caspase-1 Western blots (Fig. 1A). Interestingly, prolonged LPS stimulation reduced caspase-1 processing in a time-dependent fashion, and caspase-1 maturation was dramatically reduced in lysates of ATP-treated BMDMs that had been stimulated with LPS for 24 h (Fig. 1A). In addition to preventing caspase-1 processing, prolonged LPS treatment also reduced ATP-induced secretion of mature IL-1β and IL-18 in the culture supernatants (Fig. 1C,D).

To address whether prolonged LPS exposure specifically interfered with ATP-induced activation of the NLRP3 inflammasome, we examined its effect on caspase-1 processing by nigericin. As expected, BMDMs treated with LPS for 4 h responded with robust nigericin-induced caspase-1 processing (Fig. 1B), and secretion of IL-1β (Fig. 1C) and IL-18 (Fig. 1D). However, these responses were all dramatically blunted in macrophages that had been exposed to LPS for 12–24 h (Fig. 1A–D). To address whether the concentration of LPS mattered in regulating the NLRP3 inflammasome, we stimulated BMDMs with low (1 and 10 ng/ml) or high (100 and 1000 ng/ml) concentrations of LPS for the indicated period of times (Supplementary Fig. S1). LPS induced robust caspase-1 activation in a dose dependent manner at early time points, which were similarly reduced at chronic time points (Supplementary Fig. S1). In addition to LPS, prolonged TLR2 engagement with PAM3CSK4 also diminished ATP-induced caspase-1 activation and IL-1β release (Supplementary Fig. S2), suggesting that the regulation of NLRP3 inflammasome following chronic stimulation is not specific to TLR4 alone. All these responses were specific and required both priming (LPS/PAM3CSK4) and activation signals (ATP/nigericin), since LPS, PAM3CSK4, ATP or nigericin alone did not induce caspase-1 activation or IL-1β production (Fig. 1 and Supplementary Fig. S2). To determine whether chronic TLR stimulation affected expression of IL-1β and IL-18, we stimulated BMDMs with LPS or PAM3CSK4 for 4, 12 and 24 h and assessed the levels of pro-IL1β and pro-IL-18 by Western blot. Our experiments showed that LPS or PAM3CSK4 stimulation for 24 h reduced the levels of pro-IL-1β, however pro-IL-18 levels increased in a time dependent manner (Supplementary Fig. S3). These results show that chronic TLR2 or TLR4 stimulation can directly regulate IL-1β levels, however, IL-18 levels are regulated through inflammasome-mediated processing.

### Secreted factors are responsible for chronic LPS-induced control of NLRP3 inflammasome activation

**Figure 2:**
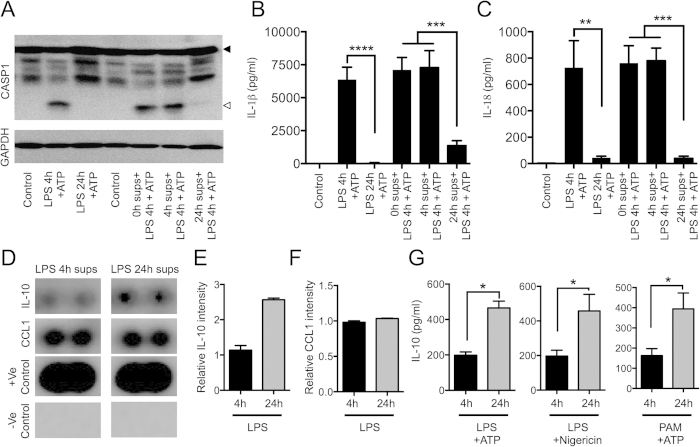
Secreted factors produced during chronic LPS stimulations reduce NLRP3 inflammasome activation. (**A–C**) Control supernatant, 4 h supernatant and 24 h supernatants from LPS stimulated samples were added to fresh BMDMs and stimulated with LPS/ATP for 4 h. Caspase-1 activation (**A**), IL-1β release (**B**) and IL-18 release (**C**) were determined. (**D–F**) WT BMDMs were stimulated with LPS for 4 or 24 h and supernatants were collected. IL-10 and CCL1 in the 4 and 24 h supernatants were determined by cytokine profiler reverse Western kit (R&D systems). (**D**) IL-10 and CCL1 blots for 4 and 24 h supernatants. (**E**) IL-10 band intensity normalized to 4 h IL-10 levels. (**F**) CCL1 levels normalized to 4 h CCL1 levels. (**G**) BMDMs were stimulated with LPS or PAM3CSK4 for 4 and 24 h followed by ATP/nigericin for the last 30 minutes. IL-10 in the supernatants of stimulated samples was determined by ELISA. Solid arrow represents pro-form and open arrow represents cleaved-form of the protein in Western blots. ELISA data represent means ± s.e.m. and data are cumulative of at least three-five independent experiments. All other data are representative of at least three independent experiments. Statistical significance was determined by Student’s t test. *p < 0.05, **p < 0.01, ***p < 0.001 and ****p < 0.0001. Uncropped blots of represented western blots are presented in Supplementary Fig. 11 and 12.

Type I IFNs have been suggested to inhibit NLRP3 inflammasome^23^. Our studies showed that caspase-1 activation and IL-1β production were reduced to the same extent in chronic LPS-stimulated *Ifnar*^*−/−*^ BMDMs (Supplementary Fig. S4). Caspase-11 has also been shown to be involved in regulating NLRP3 inflammasome^24^. Stimulation of WT and *Casp11*^*−/−*^ BMDMs further demonstrated that caspase-11 is not involved in chronic LPS-mediated dampening of NLRP3 inflammasome (Supplementary Fig. S5). LPS stimulation leads to upregulation of both intracellular and secreted factors. To investigate the mechanism by which chronic LPS exposure limits potent NLRP3 inflammasome activation, we examined whether factors in the culture supernatants of BMDMs treated with LPS for 24 h could prevent NLRP3 inflammasome activation by naïve macrophages. As expected, macrophages treated with LPS for 4 h followed by ATP stimulation in fresh culture medium resulted in robust caspase-1 activation and pronounced secretion of high levels of IL-1β and IL-18. These inflammasome readouts were markedly reduced when cells were treated similarly in culture medium of BMDMs that have been exposed to LPS for 24 h, but not with culture medium of unstimulated or acute LPS-treated BMDMs (Fig. 2A–C). These results suggested that chronic LPS stimulation induces extracellular release of a soluble factor that limits NLRP3 inflammasome activation in naïve macrophages.

IL-10 is a well-established regulatory cytokine that suppresses and controls inflammation^25^. Furthermore, secretome analysis of supernatants from LPS-stimulated macrophages revealed IL-10 to be secreted with prolonged, but not short LPS treatment^26^. More importantly, study of *Il10*^*−/−*^ BMDMs suggested that IL-10 regulates pro-IL-1β expression^23^. Additionally, studies with *Il10*^*−/−*^ mice and neutralizing IL10R signaling in WT mice showed enhanced expression of NLRP3 inflammasome components in an antigen-induced arthritis model^27^. To investigate whether IL-10 was involved in chronic LPS stimulation-induced suppression of the NLRP3 inflammasome, we first examined the levels of IL-10 in the supernatants of BMDMs stimulated for 4 and 24 hours. We found that the production of IL-10 increased in a time-dependent manner (Fig. 2D–G and Supplementary Fig. S6). Importantly, PAM3CSK4 stimulation also upregulated secreted levels of IL-10 with prolonged stimulation, which indicates that this is not specific to LPS (Fig. 2G and Supplementary Fig. S6A). Interestingly, chronic LPS stimulation also significantly increased IL-10R expression (Supplementary Fig. S6B–D). Collectively, these results imply that IL-10 may contribute to negative regulation of the NLRP3 inflammasome upon chronic TLR stimulation.

### IL-10 negatively regulates NLRP3 inflammasome activation during chronic LPS treatment

**Figure 3:**
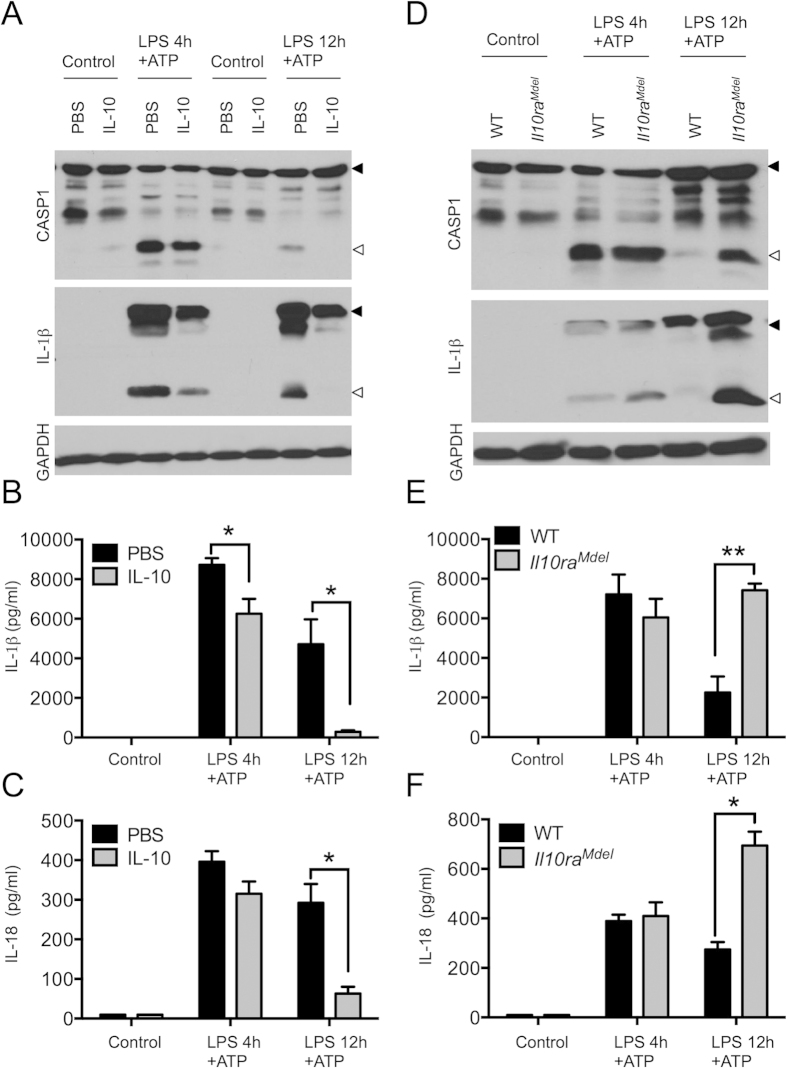
IL-10 negatively regulates NLRP3 inflammasome activation. (**A–C**) WT BMDMs were pretreated with recombinant murine IL-10 for 30 minutes followed by LPS for indicated hours (4 h, 12 h) and ATP for the last 30 minutes. (**A**) Cell lysates were immunoblotted for caspase-1, IL-1β and GAPDH. Cell supernatants were collected and analyzed for IL-1β (**B**) and IL-18 (**C**) by ELISA. (**D**–**F**) WT and *Il10ra*^*Mdel*^ (Macrophage specific deletion of IL-10R) BMDM were plated and stimulated with LPS for 4 h and 12 h followed by ATP for the last 30 minutes. (**D**) Caspase-1 activation, IL-1β cleavage, and GAPDH expression were determined in the cell lysates by Western blot. Cell supernatants were collected and analyzed for IL-1β (**E**) and IL-18 (**F**) by ELISA. Solid arrow represents pro-form and open arrow represents cleaved-form of the protein in Western blots. ELISA data represent means ± s.e.m. from three-five independent experiments and all other data are representative of at least three independent experiments. Uncropped blots of represented Western blots are presented in Supplementary Fig. 11.

To ascertain if the enhanced IL-10 production associated with chronic LPS stimulation is responsible for tempering of NLRP3 inflammasome activation, BMDMs were pre-incubated with recombinant IL-10 prior to stimulation with LPS. Addition of IL-10 prior to acute LPS/ATP stimulation significantly reduced, but did not completely abolish, the levels of processed caspase-1 and secreted IL-1β and IL-18 in macrophages (Fig. 3A–C). Dramatic reduction of NLRP3 inflammasome activation by recombinant IL-10 was also observed when cells were primed with LPS for 12 h prior to ATP stimulation (Fig. 3A–C). In addition to reducing IL-1β cleavage, IL-10 also reduced the levels of pro-IL-1β suggesting a negative regulatory role for IL-10 in priming (Fig. 3A). Thus, our results suggest that IL-10 production upon chronic LPS exposure suppresses NLRP3 inflammasome activation. To further characterize the role of IL-10 in dampening the NLRP3 inflammasome, we utilized *Il10ra*^*Mdel*^ mice that specifically lack IL-10R expression in macrophages^28^. As shown before, wild type BMDMs treated with LPS for 12 h were significantly impaired in their ability to induce caspase-1 processing and IL-1β cleavage in response to ATP. In marked contrast, prolonged LPS treatment failed to dampen NLRP3 inflammasome-mediated activation of caspase-1 and IL-1β cleavage in *Il10ra*^*Mdel*^ BMDMs (Fig. 3D). In agreement, *Il10ra*^*Mdel*^ BMDMs treated with LPS/ATP for 12 h secreted amounts of IL-1β and IL-18 in their culture medium that were comparable to those of macrophages treated with LPS/ATP for 4 h (Fig. 3E,F). In addition to the use of *Il10ra*^*Mdel*^ BMDMs, we also stimulated BMDMs with LPS for 4 or 12 h in the presence of IL-10 receptor-neutralizing antibodies (αIL-10R Ab) to inhibit IL-10 signaling. αIL-10R Ab treatment rescued caspase-1 activation in WT BMDMs that were treated with LPS/ATP for 12 h (Supplementary Fig. S7A). Concurrently, the levels of secreted IL-1β and IL-18 in 12 h LPS treated BMDMs were markedly increased by αIL-10R Ab treatment, and reached levels comparable to those of cells exposed to LPS for only 4 h (Supplementary Fig. S7B,C). Together, these observations suggest that IL-10 signaling negatively regulates NLRP3 inflammasome activation.

### IL-10 suppresses NLRP3 inflammasome activation by regulating caspase-8 activation and NLRP3 expression

**Figure 4:**
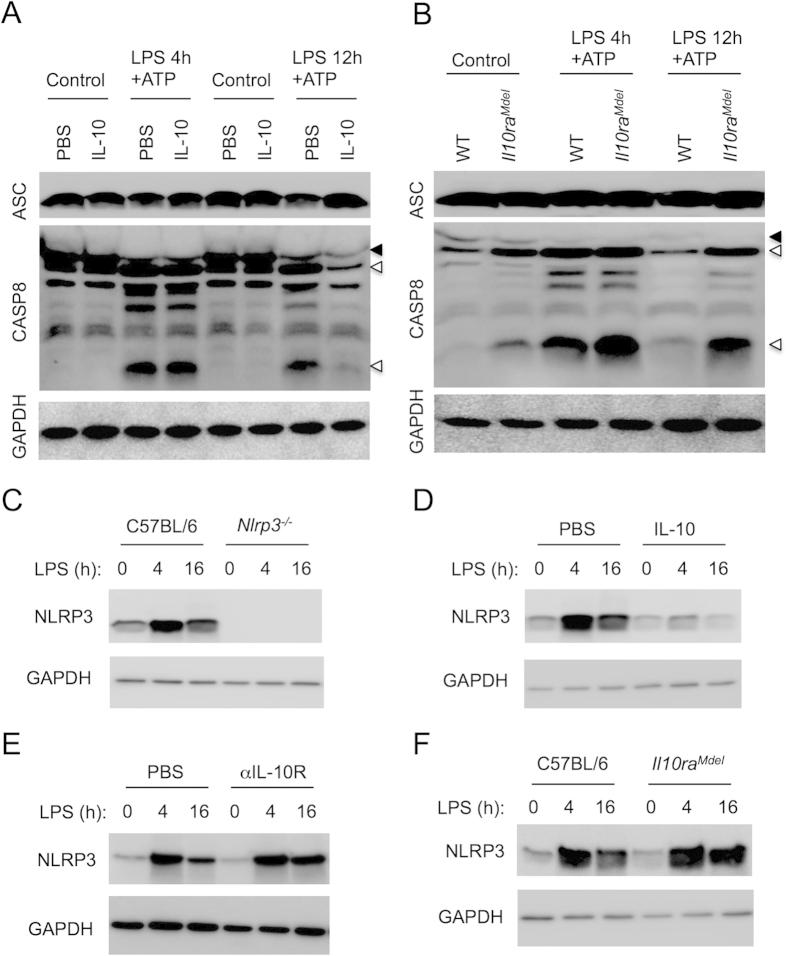
IL-10 regulates caspase-8 activation and NLRP3 levels to modulate NLRP3 inflammasome activation during chronic LPS stimulations. (**A**) WT BMDMs were pretreated with recombinant murine IL-10 for 30 minutes followed by LPS for 4 or 12 h and ATP for the last 30 minutes. Cell lysates were immunoblotted for ASC, caspase-8 and GAPDH. (**B**) WT and *Il10ra*^*Mdel*^ BMDMs were plated and stimulated with LPS for 4 and 12 h followed by ATP for the last 30 minutes. ASC, caspase-8 activation and GAPDH expression were determined in the cell lysates by Western blot. Solid arrow represents pro-form and open arrow represents cleaved-form of the protein in Western blots. (**C**) WT or *Nlrp3*^*−/−*^ BMDMs were stimulated with LPS for 4 and 16 h. NLRP3 and GAPDH expression was determined by Western blot. WT BMDMs were pretreated with recombinant murine IL-10 (**D**) or αIL-10R Ab (**E**) for 30 minutes followed by LPS for 4 or 16 h. Cell lysates were immunoblotted for NLRP3 and GAPDH. (**F**) WT and *Il10ra*^*Mdel*^ BMDM were treated with LPS for 4 and 16 h. Cell lysates were blotted for NLRP3 and GAPDH. All data are representative of at least three independent experiments. Uncropped blots of represented Western blots are presented in Supplementary Fig. 11.

Our studies demonstrate that the IL-10 signaling pathway is critical for regulating NLRP3 inflammasome activation during chronic TLR stimulations (Fig. 3). We have previously demonstrated links between the NLRP3 inflammasome and caspase-8 activity^29^. Thus, we were interested to investigate whether IL-10 abrogated caspase-1 activation by regulating caspase-8 activation. To this end, we examined caspase-8 activation in LPS/ATP stimulated BMDMs that were treated with exogenous IL-10 or impaired in IL-10R signaling (*Il10ra*^*Mdel*^). Robust caspase-8 activation was observed during acute LPS stimulations and this caspase-8 activation was reduced in chronic stimulations (Fig. 4A,B). While IL-10 pretreatment did not impact caspase-8 activation at acute time points (4 h LPS), IL-10 attenuated caspase-8 activation at chronic time points (12 h LPS) (Fig. 4A and Supplementary Fig. S8A). This caspase-8 processing was specific to NLRP3 inflammasome activation since LPS or ATP stimulation alone did not induce caspase-8 activation (Supplementary Fig. S8C). Furthermore, acute PAM3CSK4+ATP stimulation also induces caspase-8 activation (Supplementary Fig. S8C). In contrast, caspase-8 activation was dramatically increased in *Il10ra*^*Mdel*^ BMDMs at both acute and chronic time points (Fig. 4B and Supplementary Fig. S8B).

Both NLRP3 and ASC are required for LPS/ATP induced caspase-8 activation^29^. We proposed that IL-10 might be regulating the expression of either ASC or NLRP3 to control caspase-8 activation. ASC expression remained unchanged during LPS stimulation in the presence or absence of IL-10 signaling, suggesting that IL-10 does not regulate ASC expression (Fig. 4A,B). To examine whether chronic LPS stimulation limits NLRP3 expression, we stimulated BMDMs with LPS for either 4 or 16 hours in the presence or absence of exogenous IL-10 and NLRP3 protein levels were assessed. Acute LPS stimulation increased NLRP3 protein levels, however, this was reduced with chronic LPS stimulation (Fig. 4C). Moreover, addition of exogenous IL-10 dramatically dampened LPS-induced NLRP3 expression following stimulation at both 4 and 16 hours (Fig. 4D). IL-10 controlled both IL-1β and NLRP3 expression by decreasing LPS-induced mRNA expression of these proteins (Supplementary Fig. S9A,B). In contrast, αIL-10R Ab treatment of WT BMDMs rescued NLRP3 expression at chronic time points (Fig. 4E). Similarly, chronic LPS stimulation did not reduce the expression of NLRP3 in *Il10ra*^*Mdel*^ macrophages (Fig. 4F). Collectively, these results indicate that chronic LPS stimulation-induced IL-10 dampens NLRP3 inflammasome activation in part by regulating the NLRP3 expression and caspase-8 activation.

## Discussion

NLRP3 is one of the best-studied Nod-like receptors that forms an inflammasome, a multimeric protein complex that mediates processing of pro-IL-1β and pro-IL-18 into their mature forms^30^. NLRP3 inflammasomes are assembled in response to a wide array of stimuli including bacteria, viruses and fungi; as well as danger-associated signals such as ATP and nigericin. Infection or stimulation of cells ultimately results in K^+^ efflux and Ca^2+^ mobilization that activates the NLRP3 inflammasome^31^,^32^. Although studies characterizing the activation of NLRP3 inflammasomes are abundant, the factors regulating the NLRP3 inflammasomes are less clear. While several studies have suggested that IL-10 can inhibit NLRP3 inflammasome^23^,^27^,^33^, the dynamics of acute and chronic TLR stimulation and how that impacts NLRP3 inflammasome was less clear. Herein, we demonstrate that IL-10 works in a negative feedback loop to regulate NLRP3 inflammasome activation during chronic stimulations (Supplementary Fig. S10).

Previous work has shown that IFN-β induces IL-10 production through the IFNAR signaling pathway that involves STAT1^23^. Upstream of IFN-β, the TLR-TRIF signaling axis is critically involved in IFN-β production^34^. Thus, it could be argued that TLR-induced IFN-β production is responsible for induction of IL-10 synthesis through the IFNAR signaling axis. However, we see negative regulation of NLRP3 inflammasome during chronic PAM3CSK4 stimulation (Supplementary Fig. S2), which does not engage the TRIF pathway. Furthermore, chronic LPS stimulation induces similar reduction of caspase-1 activation and IL-1β production in *Ifnar2*^*−/−*^ BMDMs (Supplementary Fig. S4). This suggests that TLR-stimulation induced IL-10 and control of NLRP3 inflammasome is independent of the IFNAR signaling axis.

IL-10 has previously been shown to promote endotoxin tolerance in macrophages, where tolerized macrophages fail to produce proinflammatory cytokines upon re-challenge^25^,^35^. While we have only shown that chronic TLR-2 and TLR-4 stimulation induces IL-10 and regulates NLRP3 inflammasome, other studies have shown that IFNAR signaling, especially IFNβ induces IL-10^23^. Thus, it is possible that chronic stimulation through another TLR that engages TRIF (TLR-3) might also abrogate the NLRP3 inflammasome. IL-10 is a regulatory cytokine that is known for its negative role in inflammation. As such, *Il10*^*−/−*^ mice exhibit hyperinflammation and are susceptible to inflammatory bowel disease^36^. IL-10 has also been reported to inhibit NFκB and MAP kinases, and thus control inflammation^37^,^38^. Our studies demonstrated that the ability of IL-10 to inhibit NLRP3-mediated IL-1β secretion is in part due to its regulation of pro-IL-1β expression. While IL-10 did not appear to significantly alter the expression of ASC, our work further revealed a role for IL-10 in regulating inflammasome activation by regulating NLRP3 expression (Fig. 4). IL-10 induced reduction of NLRP3 could be attributed to enhanced proteasomal degradation of NLRP3, or inhibition of *Nlrp3* mRNA expression. Our studies with IL-10 pretreatment of WT BMDMs suggest that IL-10 negatively regulates mRNA expression of both *Il1b* and *Nlrp3* (Supplementary Fig. S9).

The IL-10/IL-10R signaling axis plays a central role in modulating inflammation. Genome wide association studies identified the IL-10 signaling axis as one of the important factors in conferring susceptibility to colitis^39^. In agreement, humans with loss of function mutations in either IL-10 or IL-10R develop severe spontaneous colitis^40^. Similarly, *Il10*^*−/−*^ and *Il10rb*^*−/−*^ mice develop spontaneous colitis due to uncontrolled inflammation^36^,^41^,^42^. While IL-10 production by macrophages is dispensable for progression of colitis, IL-10R signaling in macrophages is critically involved in controlling the progression of autoimmune colitis in mice^43^. A recent report suggested that uncontrolled NLRP3 inflammasome activation promotes the spontaneous colitis observed in *Il10*^*−/−*^ mice^44^. Gut microbiota and associated endotoxin are major sources of stimulation that promote inflammation and spontaneous colitis in *Il10*^*−/−*^ mice^45^. Our study herein provides molecular insights into how the IL-10/IL-10R signaling axis regulates NLRP3 inflammasome activation in macrophages during acute and chronic LPS/ATP stimulation, and helps to identify novel targets that could be exploited to treat colitis.

In conclusion, we have uncovered a novel regulatory pathway involving IL-10 in dampening NLRP3 inflammasome activation. Mechanistically, our studies demonstrate that IL-10 diminishes protein expression levels of NLRP3 and pro-IL-1β, which ultimately impacts caspase-8 activation to regulate activation of the NLRP3 inflammasome. Conceptually, our study is the first to uncover how NLRP3 inflammasomes are modulated during acute and chronic TLR stimulations. Therapeutically, IL-10 can be specifically targeted to either attenuate or activate NLRP3 inflammasome in the treatment of several autoinflammatory and infectious diseases.

## Methods

### Guideline statement

All methods used in this study are in accordance to protocols approved by St. Jude Children’s Research Hospital. All studies and experiments were conducted under guidelines and protocols approved by St. Jude Children’s Research Hospital on the Use and Care of Animals Committee.

### Mice

C57BL/6 were purchased from Jackson laboratory and bred at St. Jude Children’s Research Hospital in a specific pathogen-free animal care facility. *Nlrp3*^*−/−*3^ and *Il10ra*^*Mdel*^ 28 (macrophage specific deletion of IL-10Rα) mice have been previously described. Animal studies were conducted under protocols approved by St. Jude Children’s Research Hospital on the Use and Care of Animas.

### Macrophage differentiation and stimulation

Bone marrow-derived macrophages (BMDMs) were prepared as described previously^24^. In brief, bone marrow cells were grown in L-cell-conditioned IMDM medium supplemented with 10% FBS, 1% non-essential amino acid and 1% penicillin-streptomycin for 5 days to differentiate into macrophages. On day 5 BMDMs were seeded in 6-well cell culture plates. On the next day BMDMs were stimulated with LPS (Invivogen- LPS-SM) (20 ng/ml) or PAM3CSK4 (1 μg/ml) for the indicated hours of time followed by 5 mM ATP or 20 μM nigericin for the last 30 minutes. In some experiments, BMDMs were pretreated for 30 minutes with mouse recombinant IL-10 (50 ng/mL- Peprotech) or anti-mouse IL-10R mAb (1 μg/mL- BioxCell) before LPS/PAM3CSK4 stimulation.

In supernatant transfer experiments, supernatants from control (0 h sups), 4 h LPS treated (4 h sups) and 24 h LPS treated (24 h sups) BMDMs were transferred to fresh unstimulated BMDMs. These BMDMs were then stimulated with 20 ng/ml LPS for 4 h followed by ATP for the last 30 minutes. Caspase-1 activation was determined in the cell lysates and IL-1β and IL-18 production were determined in the supernatants by ELISA.

### Western blotting

Samples for immunoblotting were prepared by combining cell lysates with culture supernatants. Samples were denatured in loading buffer containing SDS and 100 mM DTT and boiled for 5 min. SDS-PAGE-separated proteins were transferred to PVDF membranes and immunoblotted with primary antibodies against caspase-1 (Adipogen, AG-20B-0042), pro-caspase-8 (Enzo Life Sciences, 1G12), cleaved caspase-8 (Cell Signaling Technology, D5B2), NLRP3 (Adipogen, AG-20B-0014), IL-1β (Cell Signaling Technology, D3H1Z), and GAPDH (Cell Signaling Technology, D16H11) followed by secondary anti-rabbit or anti-rat or anti- mouse HRP antibodies (Jackson Immuno Research Laboratories), as previously described^24^.

### Cytokine analysis

Concentrations of cytokines and chemokines were determined by multiplex ELISA (Millipore), or classical ELISA for IL-1β (eBioscience) and IL-18 (MBL international). ELISA data without statistical analysis were presented as mean ± s.e.m. of duplicate/triplicate technical repeats and all of these experiments were performed at least three times. For ELISA data with statistical analysis and showing significance, ELISA data were taken from three-five independent experiments.

### Flow cytometry

BMDMs were stimulated with 20 ng LPS for indicated hours. Cells were then stained with anti-IL10R-PE or Isotype-PE control antibody and analyzed by flow cytometry.

### Mouse Cytokine Array

IL-10, CCL1 and several other inflammatory cytokines were determined using mouse cytokine array kit by following manufacturer’s protocol (R&D Systems).

### Statistical Analysis

All data are represented as mean ± s.e.m. and all experiments were repeated at least two-three times before being reported.

## Additional Information

**How to cite this article**: Gurung, P. *et al.* Chronic TLR Stimulation Controls NLRP3 Inflammasome Activation through IL-10 Mediated Regulation of NLRP3 Expression and Caspase-8 Activation. *Sci. Rep.* **5**, 14488; doi: 10.1038/srep14488 (2015).

## Supplementary Material

Supplemental Figure 1-12
