# Supplementary material for: Chronic TLR Stimulation Controls NLRP3 Inflammasome Activation through IL-10 Mediated Regulation of NLRP3 Expression and Caspase-8 Activation
Source: Sci Rep. 2015 Sep 28;5:14488. doi: 10.1038/srep14488 (PMC4585974; doi:10.1038/srep14488)

SUPPLEMENTARY INFORMATION

Chronic TLR Stimulation Control NLRP3 Inflammasome Activation through IL-10 Mediated Regulation of NLRP3 Expression and Caspase-8 Activation.

Prajwal Gurung[1], Bofeng Li[2], R.K. Subbarao Malireddi[1], Mohamed Lamkanfi[3,4], Terrence L. Geiger[2] and Thirumala-Devi Kanneganti [1,*]

[1] Department of Immunology, St. Jude Children's Research Hospital, Memphis, TN, 38105, USA;

[2] Department of Pathology, St. Jude Children's Research Hospital, Memphis, TN, 38105, USA;

[3] Department of Medical Protein Research, VIB, B-9000 Ghent, Belgium;

[4] Department of Biochemistry, Ghent University, B-9000 Ghent, Belgium.

Supplementary figure legends

Supplementary Figure S1. Chronic LPS stimulation regulates NLRP3 inflammasome activation independent of LPS dose used for priming. WT BMDMs were stimulated with 1, 10, 100 and 1000 ng/ml LPS for indicated hours followed by ATP for the last 30 minutes. Cell lysates were immunoblotted for caspase-1 or actin.

Supplementary Figure S2. Chronic PAM3CSK4 stimulation induces weak NLRP3 inflammasome activation. WT BMDMs were stimulated with PAM3CSK4 for 4, 12 or 24h followed by ATP for the last 30 minutes. (**A**) Cell lysates were immunoblotted for caspase-1 and actin. (**B**) Levels of IL-1β in the supernatants were determined by ELISA. ELISA data are presented as means ± s.e.m. of technical replicates and all data are representative of at least three independent experiments.

Supplementary Figure S3. Regulation of pro-IL-1β and pro-IL-18 during acute and chronic LPS stimulation. WT BMDMs were left untreated, treated with ATP or treated with LPS/PAM3CSK4 for the indicated periods of time (0, 4 and 24 hours). The samples were then analyzed for pro-IL1β and pro-IL18 expression by western blot.

Supplementary Figure S4. IFNAR signaling axis is dispensable for chronic LPS induced attenuation of NLRP3 inflammasome. WT and *Ifnar2*$^{-/-}$ BMDMs were stimulated with LPS for 4 or 24h followed by ATP for the last 30 minutes. (**A**) Cell lysates were immunoblotted for caspase-1. (**B**) Levels of IL-1β in the supernatants were determined by ELISA. ELISA data are presented as means ± s.e.m. of technical replicates and all data are representative of at least three independent experiments

Supplementary Figure S5. Caspase-11 is dispensable for chronic LPS induced regulation of NLRP3 inflammasome. WT and *Casp11*$^{-/-}$ BMDMs were stimulated with LPS for 4 or 24h followed by ATP for the last 30 minutes. (**A**) Cell lysates were immunoblotted for caspase-1. (**B**) Levels of IL-1β in the supernatants were determined by ELISA. ELISA data are presented as means ± s.e.m. of technical replicates and all data are representative of at least three independent experiments.

Supplementary Figure S6. Chronic LPS and PAM stimulation increases IL-10 and IL-10R expression. (**A**) WT BMDMs were untreated, stimulated with LPS, PAM or LPS+ATP/PAM+ATP for the indicated hours. IL-10 in the supernatants of these BMDMs was determined by ELISA. (**B-D**) WT BMDMs were stimulated with LPS for 4 or 24 hours and expression of cell surface IL-10R was determined by flow cytometry. Data are presented as means ± s.e.m. of technical replicates and all data are representative of at least three independent experiments.****=p<0.0001, Student's t-test.

Supplementary Figure S7. αIL-10R pretreatment rescues NLRP3 inflammasome activation during chronic LPS/ATP stimulations. WT BMDMs were pretreated with αIL-10R mAb for 30 minutes followed by LPS for indicated hours (4h, 12h) and ATP for the last 30 minutes. (**A**) Cell lysates were immunoblotted for caspase-1, IL-1β, and GAPDH. Cell supernatants were collected and analyzed for IL-1β (**B**) and IL-18 (**C)** by ELISA. Solid arrow represents pro-form and open arrow represents cleaved-form of the protein in Western blots. ELISA data represent means ± s.e.m. 3-5 independent experiments and all other data are representative of at least three independent experiments.

Supplementary Figure S8. IL-10 regulates caspase-8 activation and NLRP3 levels to modulate NLRP3 inflammasome activation during chronic LPS stimulations. (**A**) WT BMDMs were pretreated with recombinant murine IL-10 for 30 minutes followed by LPS for 4 or 12h and ATP for the last 30 minutes. **(B)** WT and *Il10ra*^{Mdel} BMDMs were plated and stimulated with LPS for 4 and 12h followed by ATP for the last 30 minutes. Processed caspase-8 bands were quantified using Carestream Molecular Imaging Software program. The band intensities of untreated WT BMDMs were normalized to 1 for quantification. (**C**) WT BMDMs were untreated, stimulated with ATP alone, LPS alone, PAM3CSK4 alone or PAM3CSK4+ATP for indicated hours. Lysates were blotted for caspase-8. Data represent means ± s.e.m. and are cumulative of two-three independent experiments.

Supplementary Figure S9. IL-10 regulates mRNA expression of IL-1β and NLRP3 levels during LPS stimulations. WT BMDMs were pretreated with recombinant murine IL-10 for 30 minutes followed by LPS stimulation for 1.5, 3 and 6 hours. mRNA was isolated from these cells and expression of *Il1b* (**A**) and *Nlrp3* **(B**) were determined by quantitative RT-PCR. Data represent means ± s.e.m. and are representative of two independent experiments

Supplementary Figure S10. Chronic LPS-induced suppression of NLRP3 inflammasome activation is mediated by IL-10. <u>Acute LPS stimulation (4 hours):</u> During acute stimulations, LPS primes through TLR4 to induce upregulation of pro-IL-1β and NLRP3. ATP induces K^+ efflux and activates the NLRP3 inflammasomes. <u>Chronic LPS stimulation (12-24 hours)</u>: During chronic stimulations, LPS priming induces IL-10 production that signals through IL-10R to negatively regulate the NLRP3 inflammasome

activation. Our study demonstrated that IL-10 signaling suppresses expression of NLRP3 to limit caspase-8 activation and subsequent activation of the NLRP3 inflammasome.

Supplementary Figure S11. Uncropped blots relating to the western blots in Figures 2A, 3D, 4B, 4C, 4D, 4E and 4F.

Supplementary Figure S12. Uncropped blots relating to Figure 2D.

Supplementary Figure S1

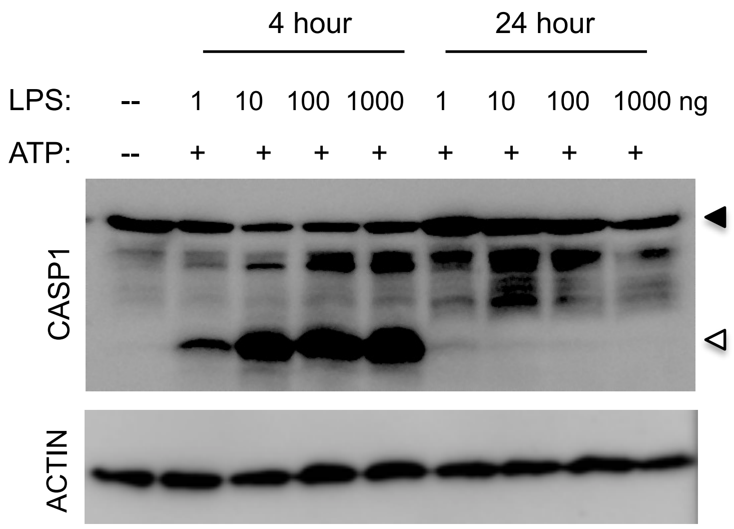

Supplementary Figure S2

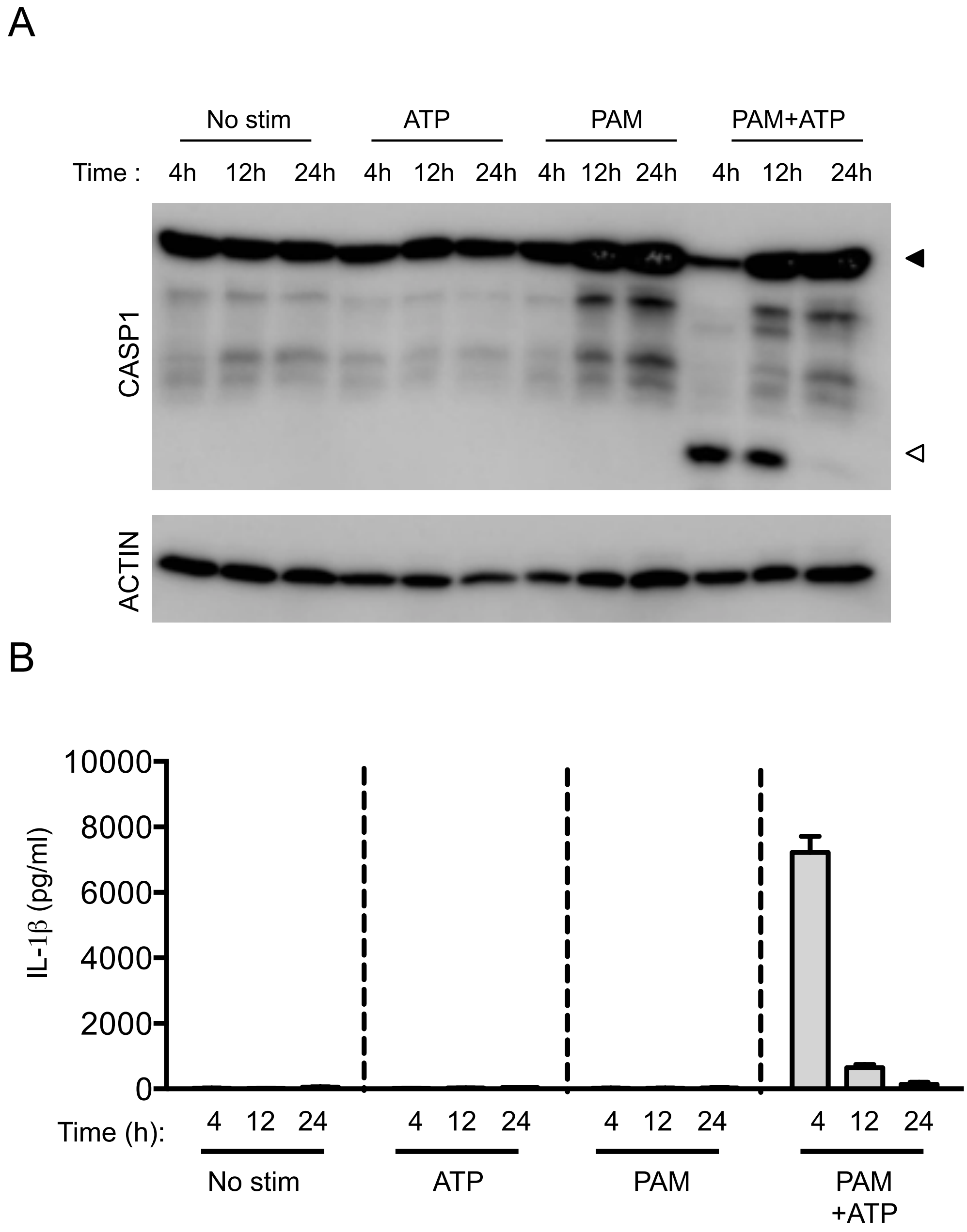

Supplemental Figure S3

A

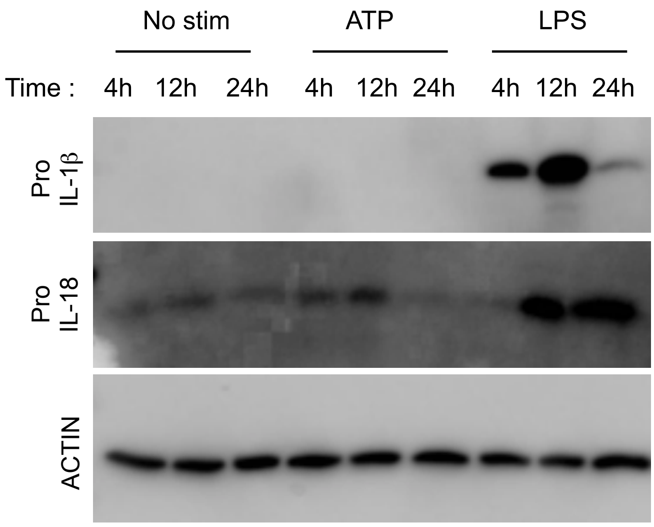

B

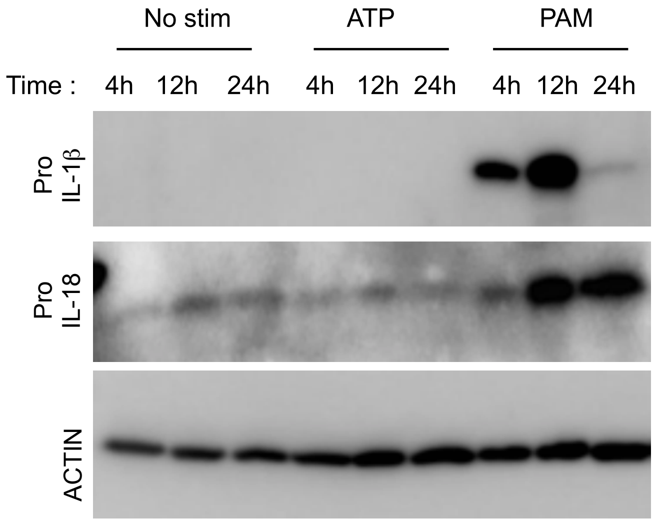

Supplemental Figure S4

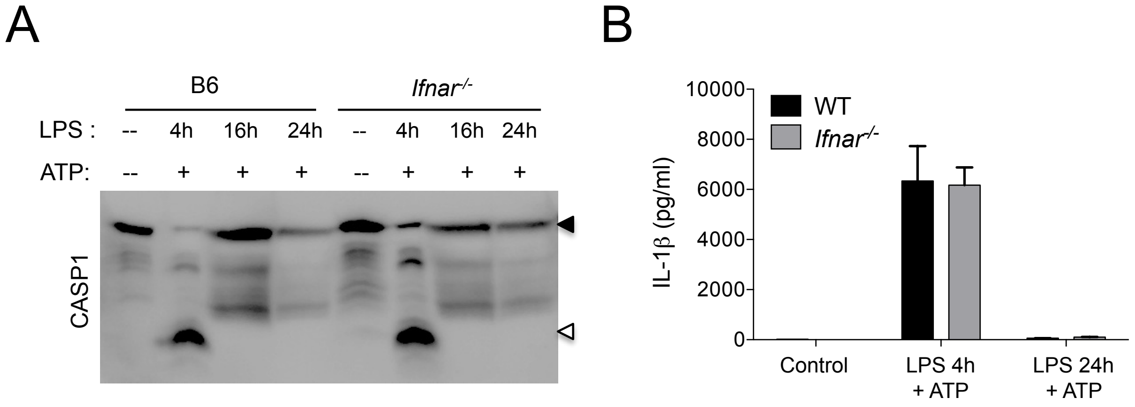

Supplemental Figure S5

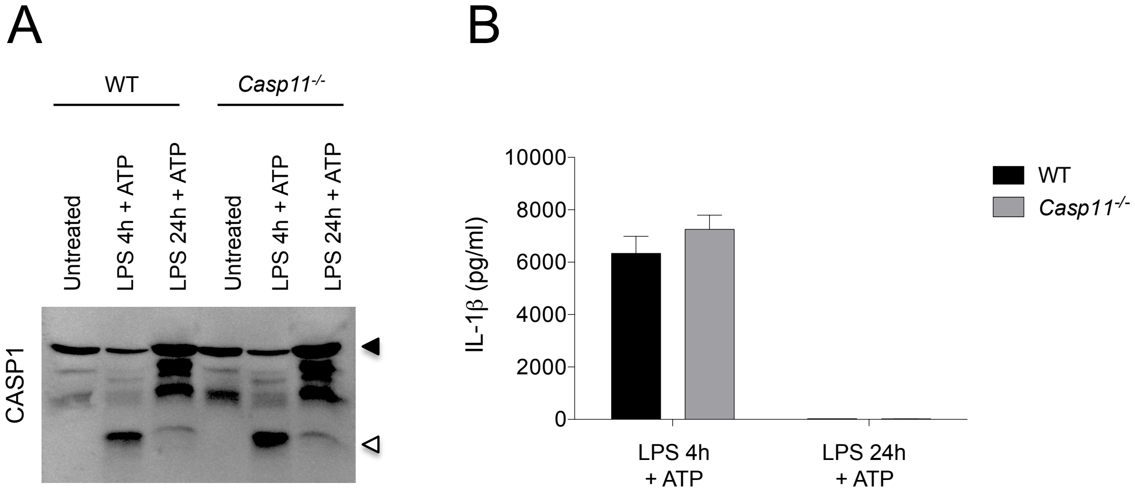

Supplemental Figure S6

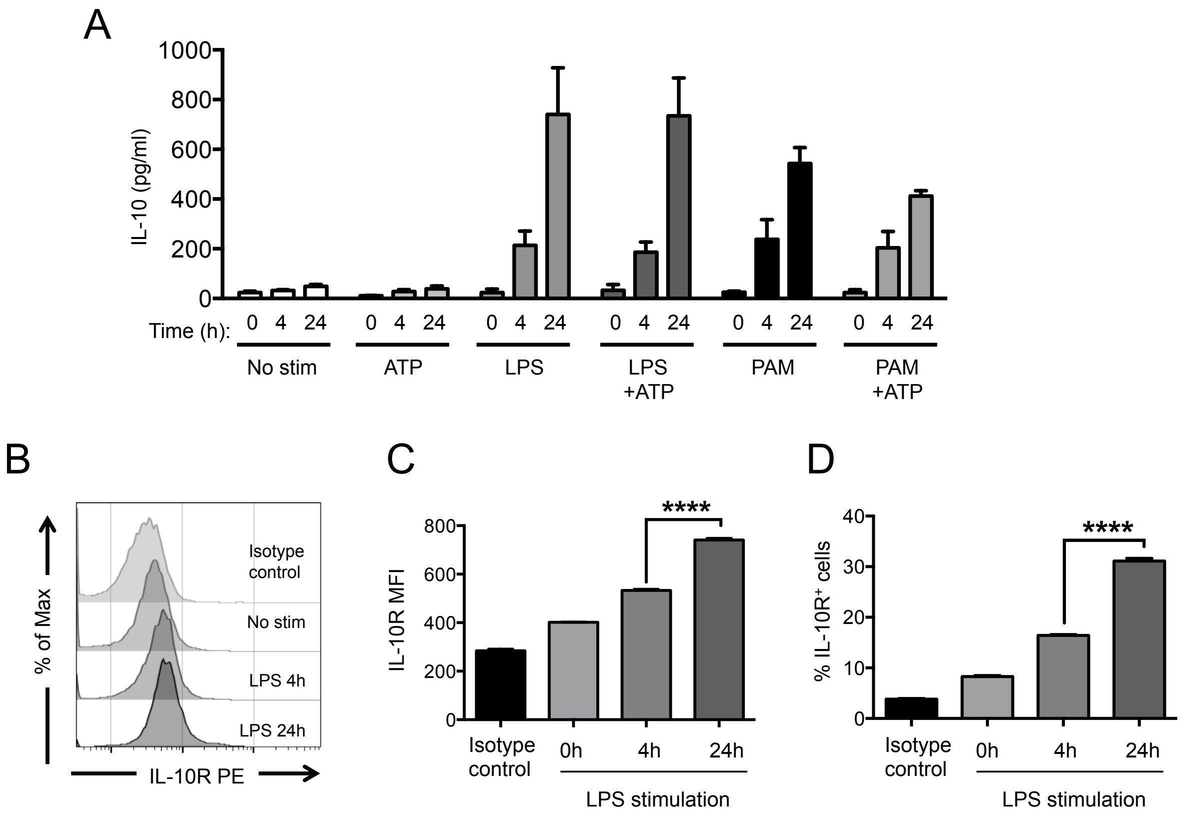

Supplementary Figure S7

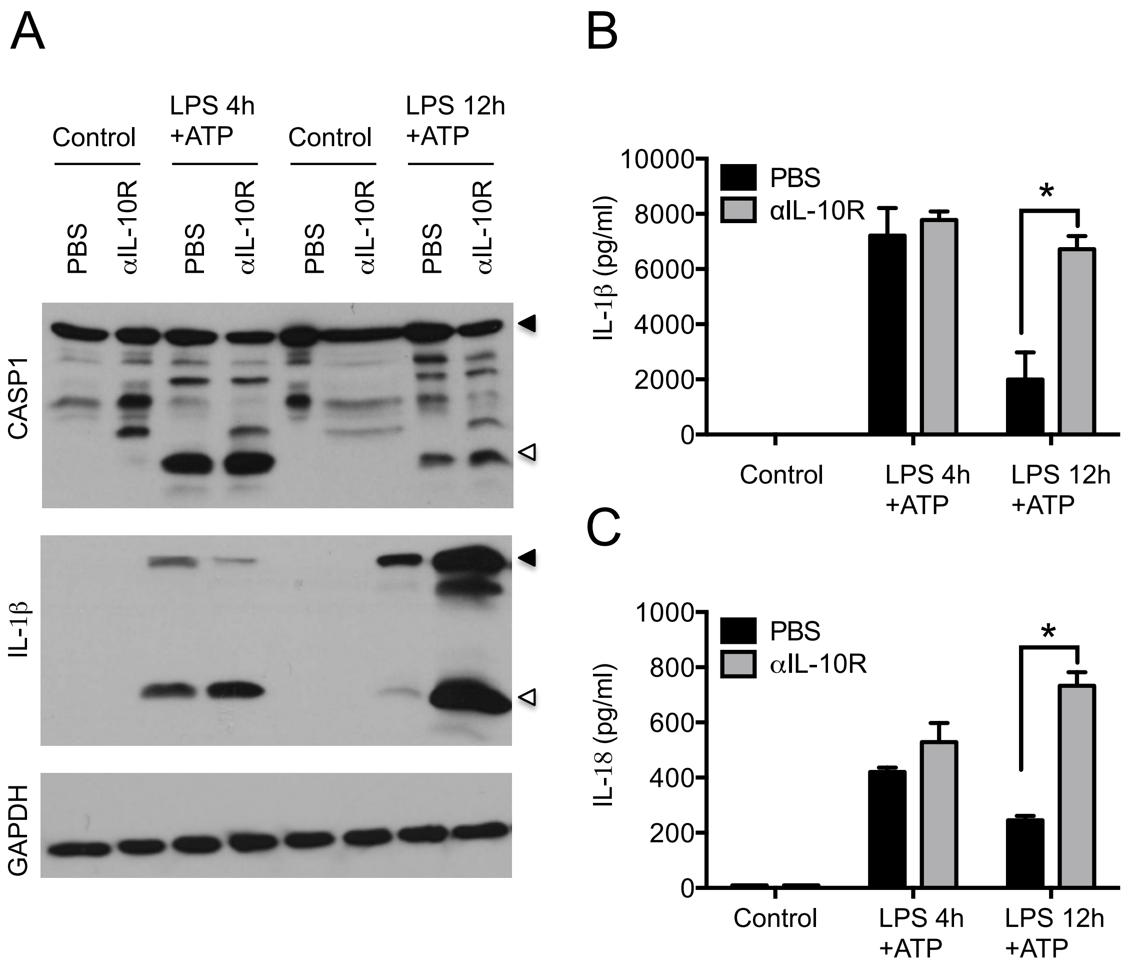

Supplementary Figure S8

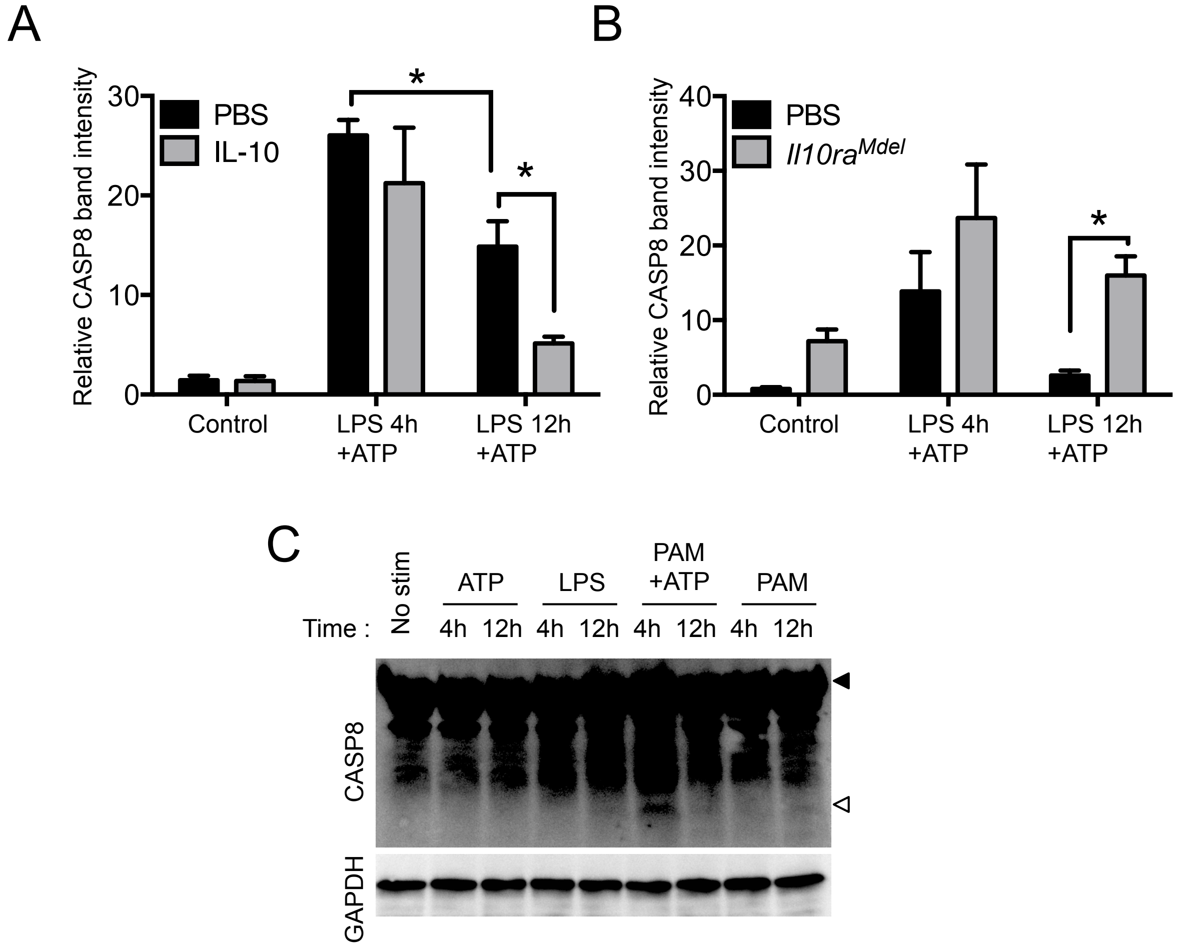

Supplemental Figure S9

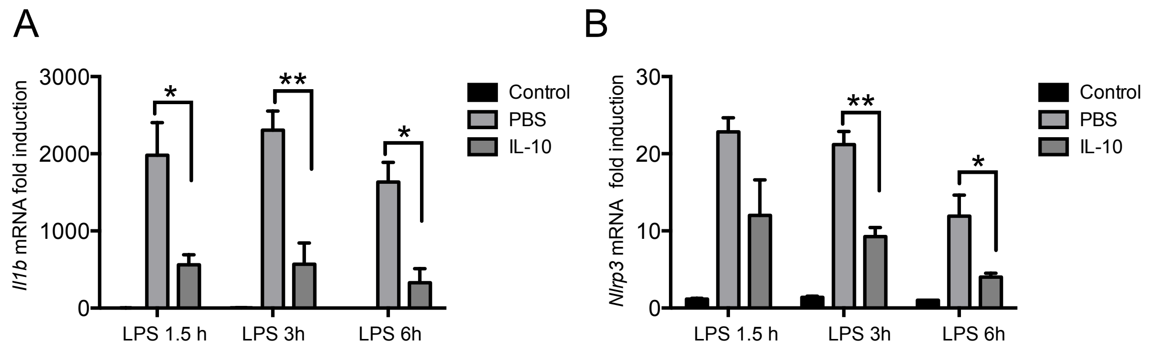

Supplementary Figure S10

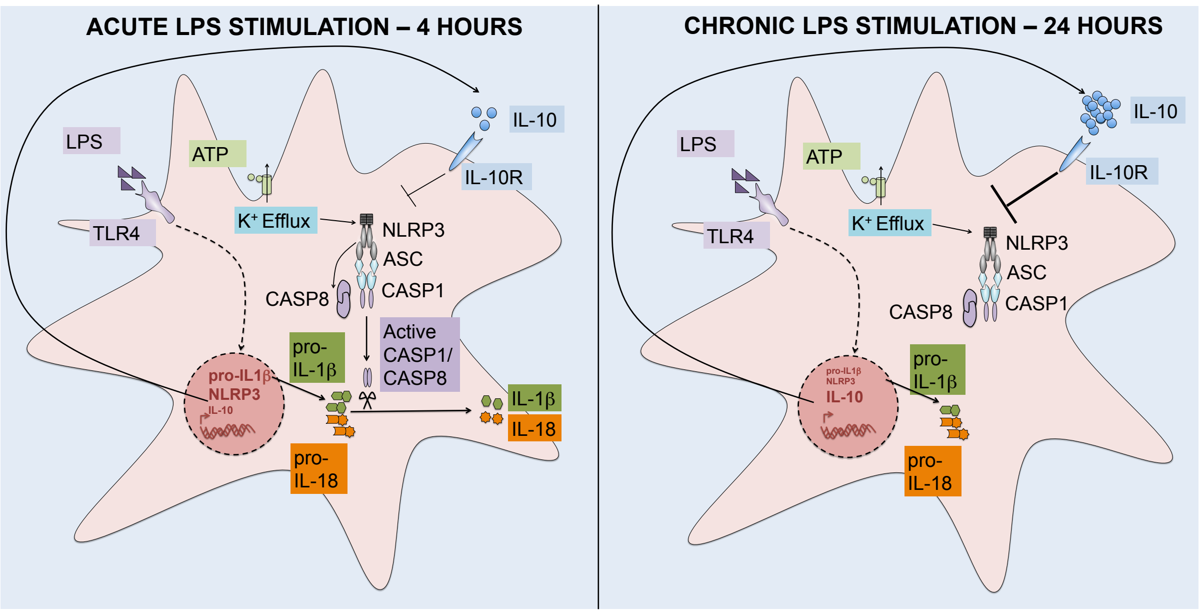

Supplementary Figure S11

A. Ucropped Fig. 2A

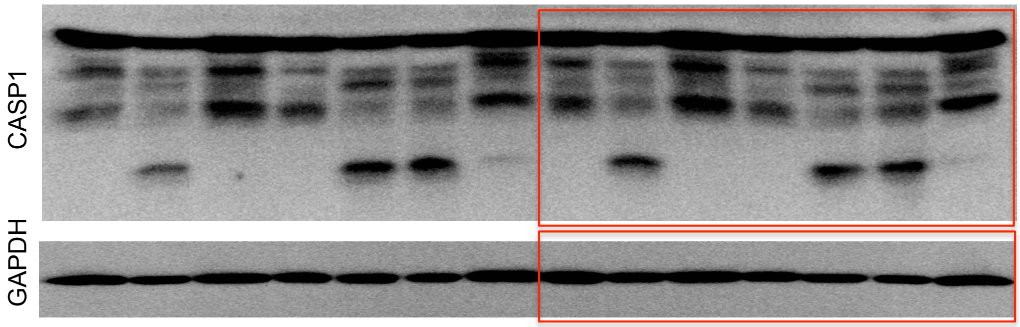

B. Ucropped Fig. 4B

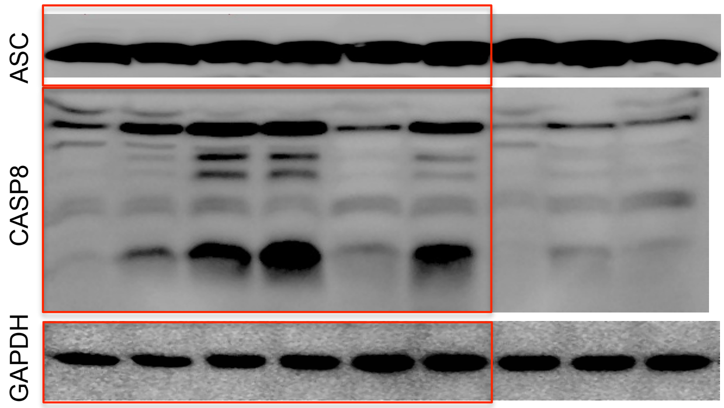

C. Ucropped Fig. 3D

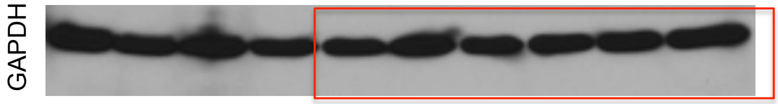

D. Ucropped Fig. 4C

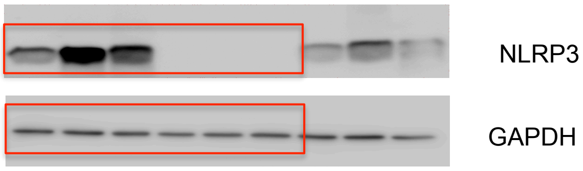

E. Ucropped Fig. 4D

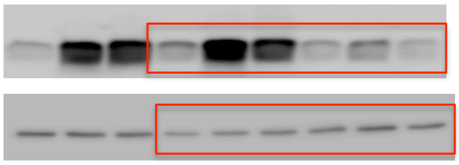

F. Ucropped Fig. 4E

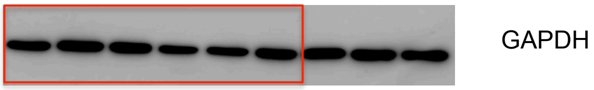

G. Ucropped Fig. 4F

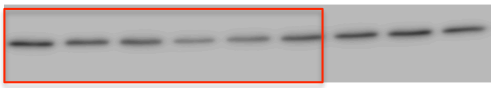

Supplementary Figure S12

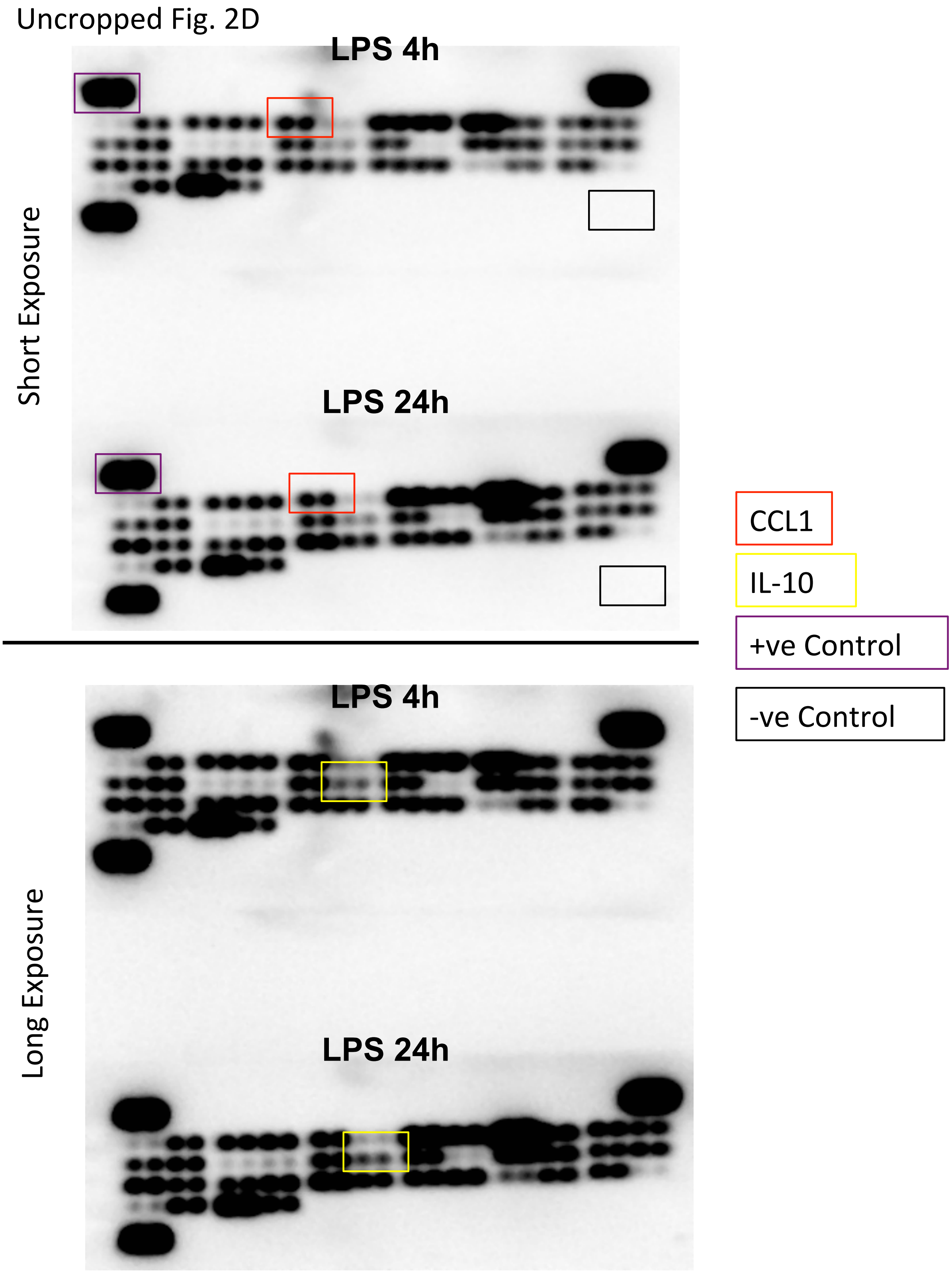

Supplement: Supplemental Figure 1-12 [file srep14488-s1.pdf]
